# Trauma-related memories in PTSD after interpersonal violence: an ambulatory assessment study

**DOI:** 10.1080/20008198.2017.1409062

**Published:** 2017-12-12

**Authors:** Nikolaus Kleindienst, Kathlen Priebe, Mirja Petri, Amélie Hecht, Philip Santangelo, Martin Bohus, Olaf Schulte-Herbrüggen

**Affiliations:** ^a^ Institute for Psychiatric and Psychosomatic Psychotherapy, Central Institute of Mental Health Mannheim, Medical Faculty Mannheim, Heidelberg University, Germany; ^b^ Department of Psychology, Faculty of Life Sciences, Humboldt-Universitaet zu Berlin, Berlin, Germany; ^c^ Department of Psychiatry and Psychotherapy, Charité-University Medicine, Berlin, Germany; ^d^ Department of Sport and Sport Science, Karlsruhe Institute of Technology, Karlsruhe, Germany; ^e^ Department of Health, University of Antwerp, Antwerp, Belgium

**Keywords:** Ambulatory assessment, childhood sexual abuse, ecological momentary assessment, intrusion, memory, posttraumatic stress disorder, trauma, violence, evaluación ambulatoria, abuso sexual infantil, evaluación momentánea ecológica, intrusión, recuerdo, trastorno por estrés postraumático, trauma, violencia, 动态评估, 童年性虐待, 生态瞬时评估法, 闯入, 记忆, 创伤后应激障碍, 创伤, 暴力, • Assessments in the everyday life of patients with posttraumatic stress disorder after interpersonal violence revealed a very high burden with traumarelated memories.• Systematic within-person variation of the methodology revealed a large impact of the sampling strategy on the recorded number of traumarelated memories and hereby clarifies why findings from previous studies were highly discrepant.• Future research should clarify whether other variables (e.g. the subjective stress related to intrusive memories) are less dependent on the methodology.

## Abstract

**Background**: Ambulatory assessment (AA) is increasingly recommended for assessing symptoms of posttraumatic stress disorder (PTSD). Previous AA studies provided new insights into the phenomenology of trauma-related memories, but also divergent findings. Notably, the range of trauma-related memories (a major target of psychotherapeutic interventions) reported in AA studies was as wide as 7.3 to 74.5 per week which might result from different methods used in these studies.

**Objective**: We aimed at assessing the frequency of trauma-related memories in PTSD related to interpersonal violence and investigated whether this frequency is dependent upon the method.

**Method**: For each patient trauma-related memories were assessed using two variants of smartphone-based AA: (1) Event-based sampling (EBS), i.e. participants entered data on each intrusive memory as it occurred; (2) Time-based sampling (TBS), i.e. participants reported the number of trauma-related memories they had experienced during the last two hours after they had been alerted by the smartphone. The numbers reported during the TBS-block were either analysed as reported by the participants or restricted to one per hour (rTBS). The impact of smartphone-assessments on trauma-related memories was assessed during a post-monitoring questionnaire.

**Results**: While trauma-related memories were frequent across assessments, the methodology had a huge impact on the numbers: EBS (median = 7) and rTBS (median = 6) yielded significantly lower weekly numbers of intrusive trauma-related memories than TBS (median = 49). Accordingly, the possibility to report unrestricted numbers of trauma-related memories clearly impacted the results. The post-monitoring questionnaire identified another source for the divergent findings: while feeling disrupted by the smartphone-assessments was unrelated to the numbers reported during EBS, feeling disrupted was related to an increase of trauma-related memories during TBS and rTBS.

**Conclusions**: The method clearly impacts the recorded number of trauma-related memories. Future research should clarify whether other variables (e.g. the subjective stress related to intrusive memories) are less dependent on the methodology.

## Abstract (es)

**Antecedentes:** la evaluación ambulatoria (AA) se recomienda cada vez más para evaluar los síntomas del trastorno por estrés postraumático (TEPT). Estudios previos de AA proporcionaron nuevos conocimientos sobre la fenomenología de los recuerdos relacionados con el trauma, pero también descubrimientos divergentes. En particular, la gama de recuerdos relacionados con el trauma (un objetivo principal de las intervenciones psicoterapéuticas) indicados en los estudios de AA tuvieron una amplitud de 7.3 a 74.5 por semana, lo que podría ser el resultado de los diferentes métodos utilizados en estos estudios de AA.

**Objetivo:** Nuestro objetivo fue evaluar la frecuencia de los recuerdos relacionados con el trauma en el TEPT relacionados con la violencia interpersonal e investigar si esta frecuencia depende del método.

**Método:** Para cada paciente con TEPT, los recuerdos relacionados con el trauma se evaluaron utilizando dos variantes de AA basado en teléfonos inteligentes: 1) Muestreo basado en eventos (EBS, siglas en inglés deEvent-based sampling), es decir, los participantes registraron datos sobre cada recuerdo intrusivo mientras se producía. 2) Muestreo basado en el tiempo (TBS, siglas en inglés deTime-based sampling), es decir, los participantes indicaron la cantidad de recuerdos relacionados con el trauma que habían experimentado durante las últimas dos horas después de haber recibido una señal del teléfono inteligente. Las cifras indicadas durante el bloque TBS o bien se analizaron según lo indicado por los participantes o se restringieron a uno por hora (rTBS). El impacto de las evaluaciones por teléfonos inteligentes sobre los recuerdos relacionados con el trauma se evaluó utilizando un cuestionario después de la monitorización.

**Resultados:** Aunque los recuerdos relacionados con trauma fueron frecuentes en todas las evaluaciones, la metodología tuvo un gran impacto en las cifras: EBS (mediana = 7) y rTBS (mediana = 6) arrojaron números semanales significativamente más bajos de recuerdos intrusivos traumáticos que TBS (mediana = 49). En consecuencia, la posibilidad de indicar cantidades ilimitadas de recuerdos relacionados con el trauma tuvo un claro impacto en los resultados. El cuestionario después de la monitorización identificó otra fuente de los hallazgos divergentes: Si bien sentirse interrumpido por la evaluación del teléfono inteligente no se relacionó con las cifras indicadas durante el EBS, el sentirse interrumpido se relacionó con un aumento de los recuerdos relacionados con el trauma durante TBS y rTBS.

**Conclusiones:** El método tiene un claro impacto en el número registrado de recuerdos relacionados con el trauma. La investigación futura debería aclarar si otras variables (por ejemplo, el estrés subjetivo relacionado con los recuerdos intrusivos) son menos dependientes de la metodología.

## Abstract (zh)

**背景**：动态评估（AA）是越来越受到推荐的评估创伤后应激障碍（PTSD）症状的方法。以往的AA 研究提供了关于创伤相关记忆现象的新观察，这些发现同时也是多样的。值得注意的是，在AA研究中报告创伤相关记忆的数量范围（心理治疗干预中的主要靶点）非常广泛（每周7.3到74.5次），这可能是由于这些A A研究中使用了不同的评估方法导致的。

**目标**：我们的目标是评估人际暴力相关的PTSD中创伤相关记忆的频率，并考察这种频率是否不受评估方式的影响。

**方法**：使用两种 不同的在智能手机上使用的AA对每一个PTSD 病人的创伤相关记忆进行评估：1）基于事件取样（EBS），即被试在每次闯入记忆出现时输入数据；2）基于事件取样（TBS），即当被试被智能手机提示时，报告在过去两个小时内体验到的创伤相关记忆的数量。在TBS 组，报告的数量使用被试报告量或者限制在每小时一个（rTBS）。使用事后问卷询问两种智能手机评估法对创伤相关记忆的影响。

**结果**：两种评估方法的创伤相关记忆都比较频繁，同时方法差异对报告数量有巨大影

响：EBS（中位数=7）和 rTBS（中位数=6）报告的创伤相关闯入记忆显著低于TBS（中位数=49）。相应地，可以无限制地报告创伤相关记忆显然影响了结果。事后问卷显示了另一个差异性结果的来源：虽然被智能手机干扰和EBS报告的数量无关，但和TBS 和 rTBS 中增加的创伤相关记忆有关。

**结论**：评估方法显然影响了创伤相关记忆的记录数量。未来研究应该阐明是否其它变量（比如和闯入记忆相关的主观压力）受方法影响较小。

## 1. Introduction

Involuntary memories related to traumatic events are a core symptom of posttraumatic stress disorder (PTSD) as defined in the DSM-5 (American Psychiatric Association, 2013). These memories typically occur as sensory impressions including images, body sensations, sounds/voices, or smells (Ehlers, 2015). Such memories can be highly distressing to the patients and are associated with a broad spectrum of aversive emotions that include anxiety, helplessness, anger, and sadness (Kleim, Graham, Bryant, & Ehlers, 2013; Speckens, Ehlers, Hackmann, Ruths, & Clark, 2007). Patients’ lives are further disrupted as these memories can occur at any time. Thoughts about the traumatic event were recently shifted from re-experiencing symptoms (Criterion B in the DSM-IV; American Psychiatric Association, 2000) to symptoms covering ‘negative alterations in cognitions and mood associated with the traumatic event(s)’ (Criterion D in the DSM-5; (American Psychiatric Association, 2013). This shift relates to accumulating evidence that separates sensory aspects of trauma-related memories from rumination and reflective processes with respect to phenomenology, functionality, and adequate treatment (Ehlers, 2015; Speckens et al., 2007). This distinction also impacts other symptom clusters. As such, according to the DSM-5, the intrusive and persistent re-experiencing of a traumatic event can also include nightmares and flashbacks, as well as emotional distress and physical reactions in response to a trigger.

Despite the clinical significance of trauma-related memories, even basic information such as the frequency of these memories is yet inconclusive. Inconclusiveness could be related to the assessment methods used in assessing PTSD symptoms which mostly rely on retrospective questionnaires and interviews (Chun, 2016). As a consequence, assessments of PTSD symptoms are exposed to inaccuracy and retrospective biases such as underestimating (Chun, 2016). Furthermore, some instruments only assess distress related to the symptoms and not their frequency (e.g. the PTSD Checklist for DSM-5 [PCL-5]; Weathers et al., 2013). The instruments that do take frequency into account generally use Likert scales with categories that cover broad ranges like ‘six or more times a week’ (e.g. the Posttraumatic Stress Diagnostic Scale for DSM-5 [PDS-5]; Foa et al. 2016a); Posttraumatic Stress Disorder Symptom Scale Interview for DSM-5 [PSSI-5]; Foa et al., 2016b). However, recent research (Priebe et al., 2013) points towards ceiling effects that are inherent to these commonly used questionnaires and interviews. As such, these assessments might miss capturing differences or changes in trauma-related memories in the upper range. Another drawback related to traditional assessments of PTSD symptoms is incomplete ecological validity – especially as intrusive memories are highly contextual. To overcome these drawbacks and to provide stronger ecological validity, there is a shift towards using ambulatory assessment (AA) during the usual life of the patient both for the assessment of psychopathology in general (FDA, 2009; Trull & Ebner-Priemer, 2013) and for the assessment of PTSD symptoms in particular (Chun, 2016; Walz, Nauta, & Aan Het Rot, 2014). Currently, the two major assessment strategies when using AA are (1) Time-Based Sampling (TBS), in which participants respond to signals emitted by the device; and (2) Event-Based Sampling (EBS), in which participants initiate a diary entry when a pre-specified event occurs (Fahrenberg, Myrtek, Pawlik, & Perrez, 2007).

The first study that has used AA to collect data on trauma-related memories in PTSD was carried out by Pitman et al. (1996). Wrist watches were used to alert combat veterans four times per day and to ask them to note on a paper the number of intrusive memories they had experienced during the previous four hours. On average, the patients recorded three intrusions per day which correspond to 21 intrusions per week. However, as the recording of intrusions still implied the use of paper diaries, the results from this study might have been biased by a lack of control over timely completion of the diaries which is a well-known problem with paper-and pencil diaries (Stone, Shiffman, Schwartz, Broderick, & Hufford, 2002). Modern AA studies are typically using smartphones, which allow control over data entry. Data entry can be timestamped and responses can be restricted to predefined timeframes (Kleim et al., 2013; Pfaltz, Michael, Meyer, & Wilhelm, 2013; Priebe et al., 2013). Kleim et al. (2013) asked victims of motor vehicle accidents or assaults to carry an electronic diary with them for one week and to report each intrusive memory as it occurred (EBS). Assessment was restricted to one data entry per hour. With this method, participants who had developed PTSD after the traumatic event reported a mean number of 7.3 intrusions during the week of assessment. In a study by Pfaltz et al. (2013), PTSD-patients who had experienced different traumatic events reported trauma-related symptomatology during the daytime. They used a schedule with fixed time intervals (every three hours) for data collection (TBS). For the week of assessment, the reported mean numbers of memories and of distressing thoughts related to the trauma were 17.1 and 11.8, respectively. In our own study (Priebe et al., 2013), inpatients with a diagnosis of PTSD related to childhood sexual abuse were assessed via smartphones using TBS with fixed time intervals (every two hours, six times per day). When asked about the numbers of trauma-related intrusive symptoms, the participants reported an average of 74.5 intrusions and 24.4 flashbacks during the week of assessment. In sum, the numbers of intrusive memories reported by these AA studies ranged from 7.3 to 74.5 per week. This range is clearly above the numbers of 3.0 (Speckens et al., 2007) and 4.5 intrusive memories (Hackmann, Ehlers, Speckens, & Clark, 2004) from studies based on a single retrospective rating that did not implement AA. However, the magnitude of the range within previous AA studies requires clarification.

The previously mentioned AA studies differ among themselves with respect to a multitude of factors. Besides different samples of PTSD related to different trauma types, the differences could be related to the assessment method, i.e. whether the participant is prompted by the smartphone according to a predefined schedule (TBS; Pfaltz et al., 2013; Priebe et al., 2013) or whether the participant must actively open the app in order to report each intrusive memory as it occurred (EBS; Kleim et al., 2013). Furthermore, participants could report an unrestricted number of trauma-related memories in some studies (Pitman et al., 1996; Priebe et al., 2013), while the reported number of trauma-related memories was restricted in other studies. In the study by Pfaltz et al. (2013), participants only reported about the occurrence (not numbers) of trauma-related memories. Kleim et al. (2013) restricted assessments to one assessment per hour. To our knowledge the impact of the assessment methodology, data analysis, and data collection device has not been systematically investigated yet, so its impact on the number of intrusive memories remains unclear. There is also the possibility that repeated assessments may trigger intrusive memories (Pfaltz, Michael, Grossman, Margraf, & Wilhelm, 2010). While frequent assessments had only little impact on pain ratings in patients with chronic pain (Stone et al., 2003), intrusive memories related to PTSD are easily triggered by external cues (Elzinga & Bremner, 2002) and might therefore be influenced by frequent assessment. Furthermore, Chun (2016) hypothesized that the settings in which intrusive memories are assessed may also influence the data. This is in line with our previous study (Priebe et al., 2013) that found the highest recorded number of intrusive memories occurred within a sample of patients with PTSD that were assessed during an ongoing trauma-focused psychotherapy in a residential setting. In sum, the wide range of previously reported intrusive memories per week might relate to variations in the methodology, data analysis, setting, and population under investigation. However, the current evidence does not allow for firm conclusions and further evaluation is required.

The study at hand had two major objectives. First, we aimed at estimating the frequency of intrusive memories in subjects with PTSD related to interpersonal violence. Second, we investigated whether this frequency is dependent upon some methodological aspects that might account for the high heterogeneity between previous studies. Our subjects were studied in an outpatient setting using a study-related smartphone. In addition to counting the number of distressing trauma-related memories, we also assessed the time of preoccupation with these memories. In order to study the impact of the chosen assessment method on the number of reported trauma-related memories, the assessment method was systematically varied in each study participant in a randomized order. Each participant was assessed during both a block of EBS and during a block of TBS. In addition, we calculated a variant of TBS-assessment, restricted TBS (rTBS), by limiting the number of trauma-related memories during TBS to one per hour. Data collection was complemented with a post-monitoring questionnaire about the potential impact of the assessments on trauma-related memories.

## 2. Method

### 2.1. Participants and assessments

Subjects were recruited between 2013 and 2015 from: (1) local psychotherapists in the centres, suburbs, and surroundings of four German cities: Mannheim, Ludwigshafen, Heidelberg, and Berlin; (2) from the waiting lists for psychotherapeutic treatment at the Central Institute of Mental Health (CIMH), Mannheim, and the Charité-University Medicine, Berlin; and (3) by flyers which were displayed at the CIMH and the Charité-University. As the study started before the introduction of the DSM-5, participants were eligible if they met the diagnostic criteria for PTSD according to DSM-IV (American Psychiatric Association, 2000) as assessed with the Clinician-administered PTSD scale (CAPS; Blake et al., 1995). Furthermore, the index trauma (i.e. the currently most distressing trauma) had to be related to interpersonal violence.

Participants were required to be at least 18 years old and to have the mental abilities required for the study (verbal IQ ≥ 70 as assessed by the MWT-B; Lehrl, 2005). For safety, patients in need for immediate treatment (e.g. for acute suicidality, BMI < 16, or a serious somatic condition) were not included, as well as those with a diagnosis of schizophrenia, acute substance abuse, attempted suicide during the last four months, treatment with benzodiazepines, current residential or semi-residential treatment, or ongoing trauma-focused therapy which includes exposure elements.

Co-occurring Axis I diagnoses were assessed from the Structured Clinical Interview for DSM-IV Axis I Disorders (SCID-I; First, Spitzer, Gibbon, Williams, & Benjamin, 1997). Co-occurring Borderline Personality Disorder (BPD) was assessed from the International Personality Disorder Examination (IPDE; Loranger, 1999). The global assessment of functioning was rated using the Global Assessment of Functioning (GAF; American Psychiatric Association, 2000).

Self-ratings included the Davidson Trauma Scale (DTS; Davidson et al., 1997), the Borderline Symptom List-23 (BSL-23; Bohus et al., 2009), and the Beck Depression Inventory II (BDI-II; Beck, Steer, & Brown, 1996). Traumatic childhood adversity was assessed using the Childhood Trauma Questionnaire (CTQ; Bernstein et al., 2003).

**Figure 1.**
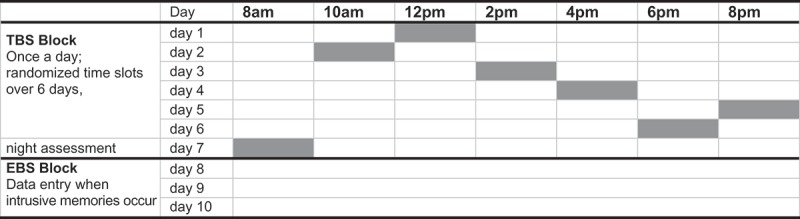
Example for a predefined assessment scheme. The assessments started with either the TBS or EBS block (randomized order). Within the TBS block the sequence of daytime assessments (at 10am, 12pm, 2pm, 4pm, 6pm, 8pm) was randomized.

The participants were provided with a smartphone (LG-P760) designated to be exclusively used for the purpose of the study. The assessments were recorded by a professional app (movisensXS for Android, https://xs.movisens.com) which was specifically programmed for this study. All other functions of a smartphone were disabled. For each participant the assessment period included both a TBS-block (seven days) and an EBS-block (three days), for details see Figure 1. The sequence of those two blocks was randomly permuted to avoid sequential effects: for half of the participants the assessments started with the TBS-block; for the other half the assessments started with the EBS-block. The study at hand focused on the frequency of intrusive memories which have been assessed during these two blocks. Data regarding further aspects of trauma-related memories including sensory perceptions involved in trauma-related memories, emotions related to these memories and potential triggers preceding these memories will be published separately.

**Figure 2.**
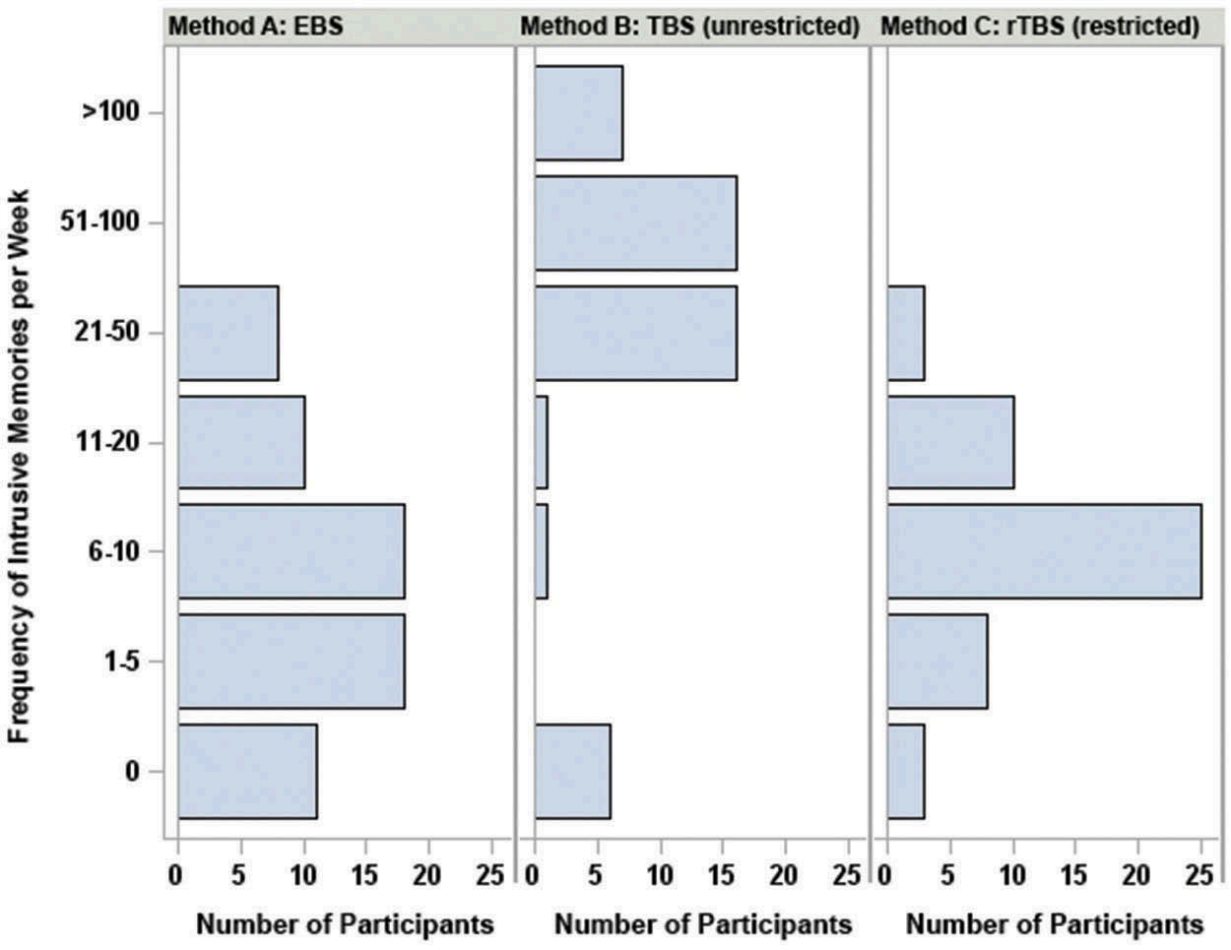
Frequencies of trauma-related memories per week as assessed by three methods: Method A: event based sampling (EBS); Method B: time based sampling (TBS) without restricting the frequency of trauma-related memories; Method C: restricted time based sampling (rTBS) limiting the frequency of trauma-related memories to one per hour.

During six days of the TBS-block the smartphone alerted the participants once during daytime (either at 10am, 12am, 2pm, 4pm, 6pm, or 8pm). The low frequency of prompts during the TBS-block of one per day was chosen to avoid reactivity. During these prompts participants were asked whether they have had trauma-related thoughts or memories during the last two hours and (if so) how many distinct episodes of trauma-related memories or thoughts they have had. In addition, they were asked how many minutes were filled with these thoughts or memories during the last two hours. Current levels of aversive inner tension for both periods with and without trauma-related memories were assessed on a visual analogue scale ranging from 0–100. During the TBS-block, the smartphone rang once in the morning (normally at 8am) during one day in order to assess intrusive memories that may have occurred during nighttime. Participants were instructed to consider only memories related to their index trauma (i.e. interpersonal violence). Trauma-related memories were defined according to the DSM-IV and included sensory memories and trauma-related thoughts. By collecting six daytime assessments from 10am till 8pm each covering two hours and the nighttime assessment covering the time from 8pm to 8am, the assessments covered the complete period of 24 hours of a day spanned across seven days. The sequence of those seven assessments was randomized. To avoid that participants would anticipate activity of the smartphone at a specific time, the randomized sequence of the timing of the assessment was concealed to the participants. The participants were only told the smartphone would ring no more than once a day between 8am and 8pm.

In contrast, during the three days of the EBS-block participants were instructed to carry the smartphone with them all the time and to start a data entry whenever an intrusive memory has occurred.

At the end of the study all participants were asked to fill out a short standardized post-monitoring questionnaire adapted from Ebner-Priemer and Sawitzki (2007) which was used to assess the participants’ experiences with the smartphone during the study. Specifically, Likert-type scales were used to assess to which extent the time with the smartphone was different than usual and whether the smartphone used for the study has altered the frequency of trauma-related thoughts or memories.

A total of 112 subjects met the inclusion criteria in the telephone interview and 83 agreed to be assessed with a diagnostic interview. Of the interviewed subjects, *n* = 66 participants met all inclusion criteria and provided their written informed consent. The study was approved by the competent ethics committee (ID: 2013-536N-MA) and registered at the German Clinical Trials Register (ID: DRKS00005705).

### 2.2. Data analysis

For the purpose of comparing our results with the results of other studies, the recorded numbers of trauma-related memories in the EBS- and TBS-blocks were expressed as numbers per week. For the TBS-block two approaches for calculating the frequency of trauma-related memories were used: (1) in line with Pitman et al. (1996) and Priebe et al. (2013), we analysed the number of distinct episodes of trauma-related memories or thoughts as reported by the participants; (2) in order to allow for comparisons with studies that did not provide the opportunity to enter an unlimited number of trauma-related memories (Kleim et al., 2013; Pfaltz et al., 2013), an alternative way to calculate the frequency of trauma-related memories was used. In line with Kleim et al. (2013) the number of intrusive memories was restricted to one per hour, i.e. memories reported at a higher frequency than one per hour were merged with the first memory and counted as a single cluster/episode. This approach which was realized during the data analyses is referred to as restricted TBS (rTBS).

Since the numbers of intrusive memories were typically skewed, the average numbers were primarily reported as medians. Non-parametric Friedman- and sign-tests were used to test the hypothesis that the frequency of trauma-related memories is dependent upon the method of assessment. Friedman-tests were used for a global comparison (EBS vs TBS vs rTBS); sign-tests were used for pairwise post-hoc comparisons. Wilcoxon signed rank tests were used to compare the levels of aversive inner tension: (1) for the 2h-periods assessed during the TBS-block which included at least one trauma-related memory vs the 2h-periods assessed during the TBS-block which included no trauma-related memories; and (2) for the 2h-periods assessed during the TBS-block which included at least one trauma-related memory vs the EBS-phase. Kolmogorov-Smirnov tests were used to compare the numbers of trauma-related memories in the subgroups of participants who did report a significant increase due to the smartphone to those who did not. *p*-values ≤ .05 (2-tailed) were considered statistically significant. Data analyses were carried out using SAS™ (v.9.4).

## 3. Results

### 3.1. Patient characteristics

A total of 66 subjects (55 female and 11 male) participated in the study. On average participants were 38.9 years old (*SD* = 11.6, range: 18–61). Pursuant to the inclusion criteria, all participants had a diagnosis of PTSD and the index trauma was related to interpersonal violence. In 47.0% of the participants the index trauma had occurred below 18 years; in 53.0% the index trauma had occurred in adulthood. With respect to the index trauma the mean age at the beginning of childhood abuse was 7.1 years (*SD* = 4.7, range 0–15 years). The mean age of the occurrence of interpersonal violence in adulthood was 32.8 (*SD* = 10.5, range: 18–60 years). The mean time elapsed since the end of the interpersonal violence index trauma was 27.3 years (*SD* = 12.8) for participants with childhood abuse and 2.5 years (*SD* = 4.9) for participants who experienced interpersonal violence in adulthood. Next to a PTSD diagnosis, the participants met on average 1.3 (range: 0–8) current Axis I diagnoses. The most prevalent co-occurring psychiatric disorders were Major Depressive Disorder (34.8%), Specific Phobia (16.7%), Panic Disorder (15.2%), Social Phobia (13.6%), Eating Disorders (10.6%), and Somatoform Pain Disorder (7.6%). In addition, 7.6% of the patients met the diagnostic criteria of BPD. One out of three participants (33.3%) reported a history of at least one suicide attempt. On average, the GAF score was 54.5 (*SD* = 9.4, range: 32–78). The mean scores for the self-ratings scales to assess psychopathology were as follows: DTS total score: 83.2 (*SD* = 24.0, range: 38–123); BSL-23 mean score: 1.6 (*SD* = 0.9, range: 0.0–3.7), and BDI-II total score: 35.9 (*SD* = 9.8, range: 18–50). The mean scores for sexual and physical abuse during childhood/adolescence as assessed from the respective scores of the CTQ were 10.9 (*SD* = 6.8, range: 5–25) and 9.9 (*SD* = 5.0, range: 5–24), respectively. The majority of participants were regularly employed (58.7%); one out of three participants was either unemployed (17.5%) or on general disability pension (17.5%); the remaining four participants (6.3%) were in vocational training. Most of the participants (80.0%) were currently in psychotherapeutic treatment but without exposure elements (mostly cognitive behavioural therapy).

### 3.2. Trauma-related memories

#### 3.2.1. EBS assessments

During the three successive days of EBS, 83.0% of the participants reported at least one trauma-related memory. If more than one trauma-related memory during the EBS-assessments was reported, the median time between two reported memories was 15 hours (mean: 17.4, *SD* = 14.1). The total number of trauma-related memories during the three days of EBS-assessments ranged from 0 to 13 which corresponds to a total of 0 to 30.3 intrusive memories in one week (median: 7, mean: 8.4, *SD* = 8.1).

#### 3.2.2. TBS assessments

During the TBS phase a total of 80.2% of the assessments were answered, speaking of an overall good compliance. The missing data entries were due to technical problems (e.g. running out of battery power) and/or incomplete data entry. During the seven TBS-assessments covering 24h, 87.2% of the participants reported at least one trauma-related memory. During the six 2h-assessments covering the daytime from 8am to 8pm, 85.1% of the participants reported at least one trauma-related memory. For the night (i.e. from 8pm to 8am), this percentage was 63.8%. The total number of trauma-related memories reported during the seven TBS-assessments covering 24h ranged from 0 to 59 (median: 7, mean: 10.1, *SD* = 11.6). This corresponds to a median number of 49 trauma-related memories per week (mean: 71.0, *SD* = 81.0). When restricting the count of trauma-related memories during the TBS-block to one per hour (rTBS), the number of intrusive memories in one week was 1–26 (median: 6, mean: 6.5, *SD* = 5.0).

As illustrated in Figure 2, the recorded weekly numbers of trauma-related memories were clearly dependent upon the method of assessment (Friedman’s Q = 52.4, *p* < .001). Post-hoc tests indicated that the numbers were similar for EBS and rTBS (*p* = .552, sign-test), but differed significantly between EBS and TBS (*p* < .001) and between rTBS and TBS (*p* < .001).

### 3.3. Frequency of trauma-related intrusive memories and aversive inner tension

During all assessments participants were asked about the current level of aversive inner tension. During the TBS-block the mean levels of aversive inner tension clearly differed between 2h-periods which were free of trauma-related memories (mean: 42.3, *SD* = 26.5, range: 0–95) and 2h-periods which included at least one trauma-related memory (mean: 64.9, *SD* = 20.6, range: 9–99). The large difference (42.3 vs 64.9) was statistically significant (Wilcoxon S = 483, *p* < .001) and indicates that a high level of distress is related to trauma-related memories. During the EBS-phase the mean level of aversive inner tension was somewhat higher (mean: 68.2, *SD* = 20.4, range: 16–98.3) and differed significantly from the 2h-periods assessed during the TBS-phase which included at least one trauma-related memory (Wilcoxon S = 184.5, *p* = .036).

### 3.4. Duration of trauma-related intrusive memories

If trauma-related memories during a daytime assessment within the TBS-block were reported, the participant was automatically asked about the duration of these memories. When summing up the duration of intrusive memories during daytime assessments of the TBS-block, the participants were preoccupied by intrusive memories for a median time of 63 minutes during the 12 hours covered by the daytime assessments (mean: 81.8, *SD* = 92.4, range: 0–585). This corresponds to a mean of 11.4% of daytime.

### 3.5. Post-monitoring questionnaire

In the post-monitoring questionnaire which was administered at the end of the study, 14.5% of the participants reported that the time with the study-related smartphone was ‘as usual’. The percentages for the categories ‘nearly as usual’, ‘somewhat different’, and ‘completely different’ were 23.6%, 36.4%, and 25.5%, respectively. With regard to a possible impact of the smartphone assessment on the frequency of trauma-related memories, 78.2% of the participants reported that the smartphone did not significantly increase the frequency of trauma-related memories (‘no impact’: 29.1%; ‘minor increase’: 34.5%; ‘minor decrease’: 10.9%; ‘significant decrease’: 3.6%). However, 21.8% reported a ‘significant increase’ of trauma-related memories due to the study-related smartphone. When comparing the subgroups of participants who did report a significant increase due to the smartphone to those who did not, there was a difference in the median of the recorded numbers for both the unrestricted TBS-phase (98 vs 46, Kolmogorov-Smirnov D = 0.525, *p* = .019) and the rTBS-phase (11 vs 6, Kolmogorov-Smirnov D = 0.518, *p* = .022). However, during the EBS-phase, both subgroups reported the same median numbers of trauma-related memories (7 vs 7, Kolmogorov-Smirnov D = 0.241, *p* = .623). Accordingly, the prompts issued by the smartphone during the TBS-phase or the anticipation of these prompts might have induced trauma-related memories in a subgroup of participants.

## 4. Discussion

We used smartphone-based AA to assess trauma-related memories during everyday life of subjects with a diagnosis of PTSD related to interpersonal violence. In order to investigate the impact of assessment strategies, the assessments during everyday life were combined with features of an experimental design, i.e. various blocks of assessment methodology were systematically manipulated in a within-subject design. During an EBS-block, participants entered data on each intrusive memory as it occurred. During a TBS-block, participants were prompted once a day and reported the number of trauma-related memories for a predefined time-frame of two hours. The numbers reported during the TBS-block were either analysed as reported by the participants or (to mirror previously used analytic strategies) restricted to one per hour (rTBS). A post-monitoring questionnaire was used to ask the participants to which extent the smartphone assessments might have altered their trauma-related memories during the study period.

In our study, the weekly numbers of trauma-related memories were similar for the EBS- and rTBS-assessments (medians of 7 and 6, respectively), but much higher for the unrestricted TBS-assessment (median = 49). Accordingly, the possibility to report unrestricted numbers of trauma-related memories for short periods apparently had a large impact on the results. Data from the post-monitoring questionnaire revealed another source possibly accounting for divergent results between EBS- and TBS-assessments: while feeling disrupted by carrying the study-related smartphone and by being assessed with this smartphone was essentially unrelated to the numbers reported during EBS, feeling disrupted was related to a marked increase of trauma-related memories during TBS and rTBS.

The study at hand confirms previous AA studies in that the number of trauma-related memories during everyday life typically exceeds the sensitive range of widely used paper-and-pencil instruments. This finding also holds true when restricting the number of reportable trauma-related memories to one per hour during the data analyses. The extremely high numbers from unrestricted TBS in our study further indicate that a substantial share of participants with PTSD after interpersonal violence experience episodes of traumatic memories very frequently. These high numbers are in line with Priebe et al. (2013) and extend their findings as our data show that such a high burden is not confined to inpatients receiving exposure-based treatment for PTSD (Priebe et al., 2013), but that it applies to outpatients who currently do not receive trauma-focused therapy. The validity of the high burden from trauma-related memories in everyday life is corroborated by the finding that our participants were preoccupied with trauma-related memories during a significant share of the daytime (11.4%) and that the level of aversive inner tension clearly increased with the presence of one or more trauma-related memories.

The systematic manipulation of the assessment methodology in a within-subject design allows attributing the differences in EBS, TBS, and rTBS numbers to the methodology. As such, the data shed light on the discrepant numbers observed in previous AA-studies. Although the limited number of previous studies precludes firm conclusions, our data are in line with the observation that studies relying on TBS (Pfaltz et al., 2013; Pitman et al., 1996; Priebe et al., 2013) reported the highest numbers, especially if the participants could report more than one trauma-related memory for the assessment interval (Pitman et al., 1996; Priebe et al., 2013). This preliminary interpretation is supported by subgroup analyses from our study indicating that the numbers during TBS- and rTBS-assessments (but not during the EBS-assessments) were increased when a participant reported being disrupted by using the study-related smartphone. This post-hoc analysis indicates that trauma-related memories might be subject to study-related reactivity when assessed with TBS. The discrepant numbers between TBS- and EBS-assessments might also relate to factors which were specific to the EBS-phase. During EBS we could not distinguish between a participant sitting at home next to the smartphone having no intrusive memory vs experiencing an intrusive memory but having the smartphone not at hand. In either case the number of trauma-related memories was counted as zero. It should also be considered that reporting distressing intrusive memories is likely aversive to some participants thus resulting in underreporting of these memories if the participants have to initiate the data entry during the EBS-phase.

All methods used in our study to assess trauma-related memories are subject to limitations. Participation in a trauma-related study and constantly wearing a study-related smartphone might lower the threshold for activation of trauma-related memories during both the TBS- and EBS-phases. Knowing that the smartphone will ring during the TBS-phase might have put some participants into a mode of alert, which likely facilitated trauma memories. We tried to minimize the impact of anticipating smartphone activity by encouraging participants not to care too much about missed prompts, by thinning out the sampling frequency to one prompt per day, and by avoiding predictability of the prompts. However, as discussed above, there is evidence that some participants felt significantly disturbed by using the smartphone and that this disturbance resulted in increased numbers of trauma-related memories reported in TBS- and rTBS-assessments. Furthermore, during TBS-assessments the participants decided whether related memories are considered as one episode or counted as separate episodes. Accordingly, the option to report unrestricted numbers for short periods was likely handled in different ways by different participants of the study, thus introducing significant between-subject variability. While the restriction of trauma-related memories during the data analyses (rTBS) partially addresses this point, the numbers calculated for rTBS tend to underestimate the numbers of trauma-related memories occurring at high frequencies. Furthermore, the numbers calculated for rTBS do not reflect the clinical phenomenon that PTSD-patients may sometimes feel deluged with trauma-related memories. As discussed above, EBS-assessments might have been subject to systematic underreporting as missed data entries were interpreted as ‘absence of trauma-related memory’ during the EBS-phase. This is a limitation inherent to the sampling scheme. On the other hand, in comparison to TBS and rTBS EBS tended to be more robust against biases resulting from feeling disturbed by using the study-related smartphone. Taken together, we are inclined to think that EBS-assessments provide a conservative and robust estimate for the number of trauma-related memories, at the expense of sensitivity. None of the currently used AA-methods for assessing intrusive memories allows for an easy interpretation. The exact method of assessment has always to be borne in mind when interpreting the numbers. A further limitation of our study relates to the high level of psychopathology and to the low level of global functioning in our sample. Accordingly, it is impossible to know whether the observed numbers of trauma-related memories can be generalized beyond the group of highly symptomatic PTSD related to interpersonal violence.

However, when considering the results of the post-monitoring questionnaire and several counter-biases, we are inclined to think that the essence of some of the findings is not challenged. Our study confirmed that the average number of distressing memories in subjects with PTSD related to interpersonal violence is outside the sensitive range of traditional assessment instruments. This raises the question whether psychotherapeutic research should be complemented by more sensitive tools to capture intrusive memories than traditional paper-and-pencil instruments. For example, the widely used Posttraumatic Diagnostic Scale (PDS-5; Foa et al., 2016a) assesses symptom frequency and severity on 5-point Likert-type scales ranging from ‘not at all’ to ‘six or more times a week/severe’. When studying effects of treatments or of specifically tailored interventions, such a lack of sensitivity might result in missing both improvements and symptom exacerbations and hereby result in an underestimation of interventions. Furthermore, our study shows that the recorded number of distressing traumatic memories highly depends on the methodology of assessment and provides first preliminary evidence for a differential susceptibility of TBS and EBS with respect to the induction of intrusive memories by the assessment method. These findings require systematic replication from an independent study. Future research should also investigate whether other variables (e.g. the subjective stress related to trauma-related memories) are less susceptible to be triggered by the assessments and whether they are less dependent on the methodology. It would be of interest to know whether the large differences between the results from the TBS-, rTBS, and EBS-assessments specifically affects the assessment of the number of intrusive memories or whether the method of assessment also affects other measures such as the subjective distress or the vividness of intrusive memories.
